# IFN-γ gene polymorphisms +874 T/A and +2109 A/G are associated with the serofast state after early syphilis treatment: a prospective observational study

**DOI:** 10.3389/fimmu.2025.1602527

**Published:** 2025-06-02

**Authors:** Konrad Kaminiów, Martyna Kiołbasa, Maciej Pastuszczak

**Affiliations:** Clinical Department of Dermatology in Zabrze, Medical University of Silesia, Katowice, Poland

**Keywords:** syphilis, serofast state, IFN-γ, polymorphism, SNP, +874 T/A, +2109 A/G

## Abstract

**Background:**

In approximately 20% of patients with early syphilis, the classical serological response pattern is absent following treatment. They experience a serofast state, which manifests as less than a 4-fold decline in non-treponemal titres, without any clinical signs of treatment failure or reinfection. The effectiveness of the immune defense against *T. pallidum*, as well as its potential failure and the occurrence of the serofast state, depends on the Th1 cellular response, including cytokines such as IFN-γ. The aim of this prospective observational study was to investigate the impact of IFN-γ gene polymorphisms on the occurrence of the serofast state.

**Materials and methods:**

A cohort of 97 patients with early syphilis (73.2% secondary syphilis, 26.8% early latent syphilis) and 50 healthy volunteers were enrolled. Two single nucleotide polymorphisms (SNPs) in the IFN-γ gene promoter region, +874 T>A (rs2430561) and +2109 A>G (rs1861494), were analyzed. Serum IFN-γ levels were measured at baseline, prior to treatment. Patients were stratified into serofast (n=18) and serologically cured (n=79) groups.

**Results:**

Serofast patients exhibited significantly lower baseline serum IFN-γ levels compared to the serologically cured group (p=0.01). All healthy subjects had IFN-γ levels below the detection limit. Analysis of IFN-γ gene polymorphisms revealed a significant association with treatment outcomes. The +874 AA and +2109 GG genotypes, associated with low IFN-γ production, were significantly more frequent in serofast patients (p=0.0004 and p=0.002, respectively), with odds ratios (OR) of 7.1 (95% CI: 2.2-23.2) and 5.5 (95% CI: 1.8-17.3), respectively. Additionally, carriers of the +874A/+2109G haplotype were significantly more likely to remain serofast (OR 4.4, p=0.01). Conversely, the +874 TT and +2109 AA genotypes, associated with high IFN-γ production, were significantly linked to serological cure (OR 4.4, p=0.03; OR 4.4, p=0.01). Similarly, the +874T/+2109A haplotype was strongly associated with serological cure (OR 17.9, p<0.0001).

**Discussion:**

Distinct IFN-γ polymorphisms and haplotypes are associated with serological outcomes in syphilis. The +874 T>A and +2109 A>G variants influence IFN-γ levels, potentially modulating the immune response and serological recovery. These findings suggest a genetic predisposition underlying serofast syphilis and underscore the importance of personalized approaches in its management.

## 1 Introduction

Syphilis, caused by the spirochete *Treponema pallidum*, remains a significant global health concern, with millions of new cases diagnosed annually. Despite the availability of effective antibiotic therapies, a subset of patients develops a serological phenomenon known as the serofast state. This condition is characterized by a less than four-fold decrease in non-treponemal antibody titres during a 6-month follow-up period after the end of treatment compared to pre-treatment values (1, 2). The pathophysiological mechanisms underlying this state remain incompletely understood, raising critical questions about whether it reflects persistent infection, residual immune activation, or other immunological phenomena (3–5).

The immune response to *T. pallidum* is predominantly mediated by Th1-driven cellular immunity, with interferon-gamma (IFN-γ) playing a central role. IFN-γ activates macrophages, enhances pathogen clearance, and coordinates downstream immune processes critical to the eradication of treponemal infection. Studies have demonstrated that higher baseline and treatment-induced IFN-γ levels are associated with favorable serological responses, including the decline of non-treponemal antibody titers following therapy (3, 4). Conversely, suboptimal IFN-γ production has been implicated in impaired pathogen clearance, potentially predisposing patients to a serofast state (3).

Emerging evidence highlights the importance of host genetic factors in modulating cytokine production and immune responses. Single nucleotide polymorphisms (SNPs) in the IFN-γ gene, particularly rs2430561 (+874 T/A) and rs1861494 (+2109 A/G), are known to regulate IFN-γ expression. The +874 T/A polymorphism, located within an intronic regulatory region, influences nuclear factor binding affinity, with the A allele associated with lower IFN-γ production (6, 7). Similarly, the G allele of the +2109 A/G polymorphism, found in the third intron, exerts a comparable suppressive effect on IFN-γ production (8, 9). Such genetic variability has been implicated in susceptibility to infectious diseases such as tuberculosis, leishmaniasis, and viral hepatitis (7, 10–13). However, its role in the immune dynamics of syphilis, particularly in the context of serofast state, remains underexplored.

Cytokine regulation in syphilis is further modulated by the interplay of pro- and anti-inflammatory mediators. For instance, elevated levels of tumor necrosis factor-alpha (TNF-α) and interleukin-10 (IL-10) have been associated with distinct immunological pathways following *T. pallidum* infection (4, 5). TNF-α is a potent pro-inflammatory cytokine critical for pathogen elimination, whereas IL-10 suppresses excessive inflammation but may inadvertently facilitate pathogen persistence. Studies have demonstrated that polymorphisms in these cytokine genes, such as TNF-α -308 G/A and IL-10–592 C/A, significantly influence the risk of serofast state by altering cytokine expression levels and immune regulation (4, 5).

Building upon previous findings by Pastuszczak et al., who demonstrated that robust pro-inflammatory responses, particularly elevated IFN-γ levels, predict serological cure in syphilis (3, 4), this study investigates the role of IFN-γ genetic polymorphisms in determining treatment outcomes. We hypothesize that specific allelic configurations associated with reduced IFN-γ production increase the risk of developing the serofast state, thereby impairing optimal immune resolution of treponemal infection.

This research aims to elucidate the genetic and immunological factors underlying the serofast state in syphilis. By bridging immunogenetic insights with clinical observations, the findings may pave the way for novel diagnostic and therapeutic approaches, enhancing the management of this complex and enigmatic condition.

## 2 Materials and methods

### 2.1 Study design

This was a prospective observational study conducted between 2018 and 2024 at the Clinical Department of Dermatology in Zabrze, Medical University of Silesia in Katowice, Poland. All consecutive patients meeting the inclusion criteria and diagnosed with early syphilis at our department between 2018 and 2024 were enrolled. No patient selection beyond the eligibility criteria was applied. Patients with early syphilis (either secondary or early latent) were enrolled during their first episode of the disease. The inclusion criteria were: age ≥18 years, positive treponemal and non-treponemal tests, no antimicrobial or immunosuppressive treatment within the 6 months preceding enrollment, no HIV infection, and no history of chronic inflammatory or autoimmune diseases. Serological diagnosis was based on reactivity in both the Rapid Plasma Reagin (RPR) test and the Treponema pallidum hemagglutination assay (TPHA). The RPR test (BD Macro-Vue™ RPR Card Test; Becton, Dickinson and Company, USA) was performed quantitatively, and the TPHA test (TPHA 200; Bio-Rad Laboratories, France) was interpreted according to the manufacturer’s guidelines, with a positive result indicating the presence of antibodies against Treponema pallidum. After enrollment, all patients received a single intramuscular dose of benzathine penicillin G (2.4 million units), in accordance with standard treatment guidelines. Healthy individuals matched for age and sex served as controls. The control group consisted of 50 healthy volunteers recruited from the hospital staff invitations. Controls were frequency-matched to the patient group by age and sex (approximately 1:2 ratio). Individuals were eligible for inclusion if they had no history of syphilis or other sexually transmitted infections, no recent (within the past 6 months) antibiotic or immunosuppressive therapy, and no chronic inflammatory or autoimmune diseases. All participants in the control group underwent a structured medical history interview to confirm eligibility. All participants were recruited at a single clinical site in Poland and represented a relatively ethnically homogeneous population, predominantly of Central or Eastern European descent.–

### 2.2 Patient characteristics

Patients with early syphilis (secondary or early latent) were enrolled in this study. Disease staging was determined according to CDC guidelines, based on clinical history, physical examination, and laboratory results (14). The diagnosis of early latent syphilis required positive treponemal and non-treponemal tests and at least one of the following within the preceding 12 months: (I) documented serologic relapse, (II) a ≥4-fold increase in non-treponemal titre, or (III) presumptive clinical evidence of primary or secondary syphilis (15). Demographic and clinical data were collected for all patients at baseline, including age, sex, and syphilis stage (secondary or early latent).

At six months post-treatment, patients were stratified into two groups according to their serological response: (I) the serofast group, defined as a <4-fold decline in RPR titre, and (II) the serologically cured group, defined as a ≥4-fold decline relative to baseline. Serofast following early syphilis treatment was defined, based on Sena AC et al. (16), as the failure to achieve at least a fourfold decline in the RPR titer six months after completion of therapy compared to the pretreatment value. Blood samples for serological testing for syphilis, including non-treponemal titre assessment, were collected at baseline and at the 6-month follow-up after treatment. In contrast, samples for cytokine level measurements and genetic analyses were obtained only at baseline.

In addition, 50 healthy volunteers aged 18 to 57 years, matched for age and sex, were recruited as a control group. None of the control participants had a history of syphilis, chronic inflammatory conditions, HIV infection or autoimmune diseases. The control group size was determined pragmatically based on the availability of eligible volunteers and was approximately matched to the patient group at a 1:2 ratio.

### 2.3 Cytokine measurement

Serum samples for IFN-γ measurement were collected at enrollment. These baseline samples were obtained prior to treatment initiation, during active syphilis infection. Samples were stored at -80°C until analysis. Serum IFN-γ levels were measured using a high-sensitivity ELISA (R&D Systems, Minneapolis, Minnesota, USA). All measurements were performed in duplicate. Cytokine measurements were performed according to the manufacturer’s instructions.

### 2.4 Genotyping for IFN-γ gene promoter polymorphisms

DNA was extracted from whole blood using the GenElute Blood Genomic DNA Kit (Sigma-Aldrich Co., St. Louis, Missouri, USA) according to the manufacturer’s protocol. SNP genotyping (+874 T/A) [rs2430561] and (+2109 A/G) [rs1861494] was determined by allelic discrimination using TaqMan SNP genotyping assays (assay IDs: C_11688847_20 and C_2683477_10; Life Technologies Co. Carlsbad, California, USA) with 7900HT Fast Real-Time PCR platform (Life Technologies).

### 2.5 Statistical analysis

Statistical analysis was performed using GraphPad Prism 7.0 software (GraphPad Software, La Jolla, California, USA). Unless otherwise noted, data were expressed as median and IQR values. The χ2 test was used to compare allele frequencies and confirm Hardy-Weinberg equilibrium. The Fisher’s exact test was used to analyze the association between serofast status and SNP variants. Continuous variables were compared using the Mann-Whitney U test. P<0.05 was considered statistically significant.

### 2.6 Ethical aspects

This study was submitted to and approved by the Jagiellonian University Bioethics Committee (approval number KBet/164/B) and by the Medical University of Silesia Bioethics Committee (approval number BNW/NWN/0052/KB1/13/I/24). Fully informed, voluntary, written consent was obtained from all study participants, including consent for genotyping.

## 3 Results

### 3.1 Characteristics of the patients

97 patients aged 18–57 years with a first episode of early syphilis, who were staged as secondary (73,5%) or early latent (26,5%) syphilis, were enrolled in this study. Six months after completing their syphilis treatment, 18 patients (18.5%) had not achieved at least a 4-fold decline in RPR titre compared with their pretreatment values. Individuals were stratified into (1) serofast state (n=18) and (2) serologically cured (n=79) groups.

The serofast state and serologically cured groups did not differ in basic demographic and clinical characteristics such as age, sex and syphilis symptoms (**Table 1**). Specific clinical signs such as macular trunk rash and palmo-plantar exanthema were listed separately to provide more detailed insight into the distribution of symptoms among patients with secondary syphilis. Patients with serofast syphilis had significantly lower baseline levels of serum IFNγ compared with individuals from the serologically-cured group (**Table 1**). All healthy subjects had IFNγ levels below the detection limit of the assay (data not shown).

**Table 1:** Characteristics of patients included in the study.

Patient Characteristic	Serofast group (n=18)	Serologically cured group (n=79)	P value
age years; (min-max)	26 (19-55)	22 (18-57)	0.5
males; n (%)	16 (89)	73 (92)	0.6
baseline RPR; (min-max)	32 (16-256)	64 (4-128)	0.4
secondary syphilis; n (%)	13 (72)	58 (73)	0.5
macular exanthema on trunk; n (%)	13 (72)	58 (73)	0.5
papular exanthema on palms and soles; n (%)	4 (22)	20 (25)	0.8
early latent syphilis; n (%)	5 (28)	21 (26,5)	0.9
pre-treatment serum IFNγ levels; pg/ml (min-max)	0.5 (0.1-2.1)	1.6 (0.1-4.3)	0.01

Data are given as median (min-max) or otherwise stated.

### 3.2 Allele frequencies of IFNγ gene promoter polymorphisms

Minor allele frequencies of the IFNγ gene +874 T>A (rs2430561, allele A) and +2109 A>G (rs1861494, allele G) polymorphisms in the whole cohort were 0.45 (for both alleles). The observed genotype frequencies agreed with Hardy-Weinberg equilibrium, both for the entire cohort.

To assess potential population stratification, we compared genotype and haplotype frequencies between syphilis patients and healthy controls. The distributions were similar across both groups. For the +874T>A polymorphism, the TT genotype was present in 30.9% of patients and 30.0% of controls, TA in 52.6% vs. 50.0%, and AA in 16.5% vs. 20.0%, respectively. For the +2109A>G polymorphism, the AA genotype was found in 49.5% of patients and 40.0% of controls, AG in 32.0% vs. 30.0%, and GG in 18.6% vs. 30.0%. The +874T/+2109A haplotype occurred in 76.3% of patients and 70.0% of controls, while the +874A/+2109G haplotype was present in 50.5% and 56.0%, respectively. None of these differences were statistically significant (all p > 0.1).

**Figure 1:**
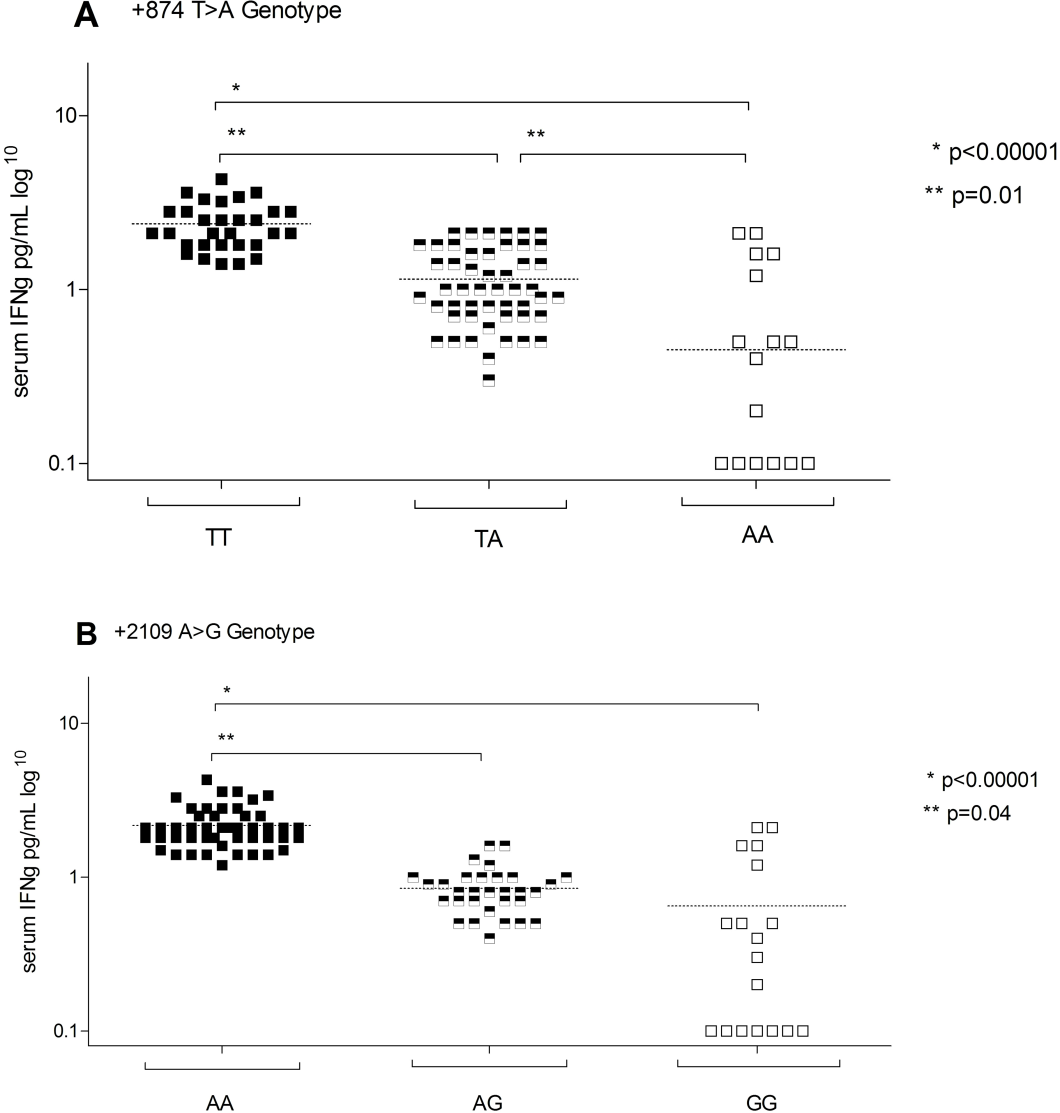
Serum IFN-γ levels in patients with syphilis according to IFN-γ gene polymorphisms.

In the whole syphilis group, individuals with the AA genotype of +874 T>A polymorphism had significantly lower pretreatment serum levels of IFNγ compared with the TA+TT genotypes (**Figure 1A**). Similarly, the GG genotype of +2109 A>G polymorphism was associated with significantly lower serum IFNγ compared with AG+AA genotypes (**Figure 1B**).

### 3.3 Association between IFNγ gene promoter SNPs and treatment outcome

There was a significant association between IFNγ gene promoter polymorphisms and the risk of serofast status after treatment. The +874T>A AA and +2109A>G GG genotypes, both associated with low IFNγ production, were significantly more frequent in patients who remained serofast compared to those who achieved serological cure (OR 7.1, 95% CI: 2.2–23.2, p=0.0004; and OR 5.5, 95% CI: 1.8–17.3, p=0.002, respectively) (**Table 2**). Similarly, carriers of the +874A/+2109G haplotype had a significantly higher likelihood of remaining serofast (OR 4.4, 95% CI: 1.3–14.6, p=0.01).

**Table 2:** Frequencies of IFNγ gene single nucleotide polymorphisms (+874 T>A and +2109 A>G) in serofast and serologically cured group.

Genotype/Haplotype	Serofast group (n=18)	Serologically cured group (n=79)	P value	OR (95%CI)
+874 T>A genotype; n (%)				
TT	2 (11.1)	28 (35.4)	0.04	0.2 (0.1-1.1)
TA	8 (44.4)	43 (54.4)	0.4	0.7 (0.2-1.9)
AA	8 (44.4)	8 (10.1)	0.0004	7.1 (2.2-23.2)
+2109 A>G genotype; n (%)				
AA	4 (22.2)	44 (55.7)	0.01	0.2 (0.1-0.8)
AG	6 (33.3)	25 (31.6)	0.9	1.1 (0.4-3.2)
GG	8 (44.4)	10 (12.7)	0.002	5.5 (1.8-17.3)
+874 T/+2109A haplotype; n (%)	5 (27.8)	69 (87.3)	0.00001	0.1 (0.02-0.2)
+874 A/+2109G haplotype; n (%)	14 (77.8)	35 (44.3)	0.01	4.4 (1.3-14.6)

OR; Odds ratio and 95%CI; 95% confidence intervals indicating the likelihood of serofast status in patients carrying a given genotype/haplotype, compared to all other genotypes combined.

In contrast, the +874T>A TT and +2109A>G AA genotypes (high IFNγ producers) were associated with a reduced risk of serofast status (OR 0.23, 95% CI: 0.05–0.82, p=0.03; and OR 0.23, 95% CI: 0.07–0.76, p=0.01, respectively). The +874T/+2109A haplotype conferred a particularly strong protective effect against the serofast state, with an OR of 0.06 (95% CI: 0.02–0.19, p<0.0001).

**Table 3** summarizes pre-treatment IFNγ concentrations stratified by clinical outcome and genotype. Across both +874T>A and +2109A>G polymorphisms, patients who achieved serological cure tended to exhibit higher IFNγ levels compared to those who remained serofast. This trend was also observed at the haplotype level, although the differences did not reach statistical significance.

**Table 3:** Pre-treatment IFN-γ serum levels in serofast and serologically cured group, depending on genotype and haplotype.

Genotype/Haplotype	Serofast group (n=18)	Serologically cured group (n=79)	P value
IFN-γ +874 T>A (pg/mL)			
TT	1.9 (1.6-2.1)	2.3 (1.4-4.3)	0.3
TA	1.2 (0.5-1.6)	1 (0.1-2.1)	0.9
AA	0.1 (0.1-0.5)	1.4 (0.5-2.1)	0.06
IFN-γ +2109 A>G (pg/mL)			
AA	1.4 (0.5-2.1)	2.1 (1.4-4.3)	0.3
AG	1.3 (0.5-1.6)	0.8 (0.4-1.0)	0.8
GG	0.1 (0.1-0.5)	0.9 (0.1-2.1)	0.1
+874 T/+2109A haplotype	1 (0.5-2.1)	1.8 (0.5-4.3)	0.6
+874 A/+2109G haplotype	0.5 (0.1-1.6)	0.8 (0.1-2.1)	0.7

Data are given as median (min-max).

## 4 Discussion

The immune response to *T. pallidum* infection has long been recognized as widespread and complex, with cellular immunity playing a pivotal role in the pathophysiology of syphilis. In early stages of the disease, cellular immune mechanisms facilitates bacterial clearance at the site of entry, often resulting in spontaneous resolution of symptoms (17). The Th1-type response is particularly prominent, and individuals exhibiting a robust Th1 profile are more likely to control infection and limit disease progression (18, 19).

Conversely, impaired cellular immunity may lead to ineffective bacterial clearance, persistent inflammation, and the development of the serofast state after treatment (3, 4, 15). Several studies suggest that an exaggerated regulatory immune response, especially mediated by interleukin-10 (IL-10), may contribute to the persistence of the serofast condition. Elevated IL-10 levels have been proposed as markers of disease activity in syphilis, particularly among people living with HIV who may display altered immune regulation due to co-infection (20). Similarly, in neurosyphilis, cerebrospinal fluid IL-10 has been identified as a potential biomarker in both symptomatic and asymptomatic patients, and is thought to facilitate bacterial persistence (21). Notably, persistently elevated IL-10 levels, both before and after treatment, have been associated with a higher likelihood of developing the serofast state (4).

Our findings, showing increased serum IFN-γ levels and associations with specific IFN-γ genotypes, are in line with recent immunopathological models highlighting the role of IFN-γ in promoting the opsonophagocytosis of *T. pallidum* by macrophages via CD64-mediated mechanisms (22). In contrast, patients exhibiting a stronger pro-inflammatory profile—characterized by higher levels of IFN-γ, tumor necrosis factor-α (TNF-α), and interleukin-6 (IL-6)—showed a greater likelihood of achieving serological cure (3, 4).

The precise mechanisms underlying the serofast state remain unclear. Possible explanations include persistent *T. pallidum* antigenic stimulation, immune dysregulation, or incomplete bacterial eradication (17). This ambiguity has significant clinical implications, as it remains uncertain whether serofast individuals are at risk of developing late-stage syphilis or pose an epidemiological threat to others (17).

In our study, 19.5% of patients exhibited a serofast response six months post-treatment. This aligns with previous reports, where the prevalence of serofast status ranged from 9.4% to 44.4% (14, 19). This broad variability may be partially attributable to differences in infection stage at the time of diagnosis and treatment. Notably, early latent syphilis has been associated with an increased likelihood of serofast status compared to primary or secondary syphilis (23).

The host immune response to *T. pallidum* involves a balance between pro- and anti-inflammatory cytokines, enabling bacterial clearance while minimizing tissue damage (17). Interferon-gamma (IFN-γ), a key pro-inflammatory cytokine, plays a pivotal role in the immune response to *T. pallidum*. Produced predominantly by T cells and natural killer (NK) cells, IFN-γ enhances T-cell cytotoxicity and macrophage-mediated bacterial phagocytosis (18). Prior studies have indicated that variations in serum IFN-γ levels may influence infection outcomes, with lower concentrations potentially facilitating bacterial persistence, chronic inflammation, and the development of a serofast state post-treatment (3, 4).

**Figure 2:**
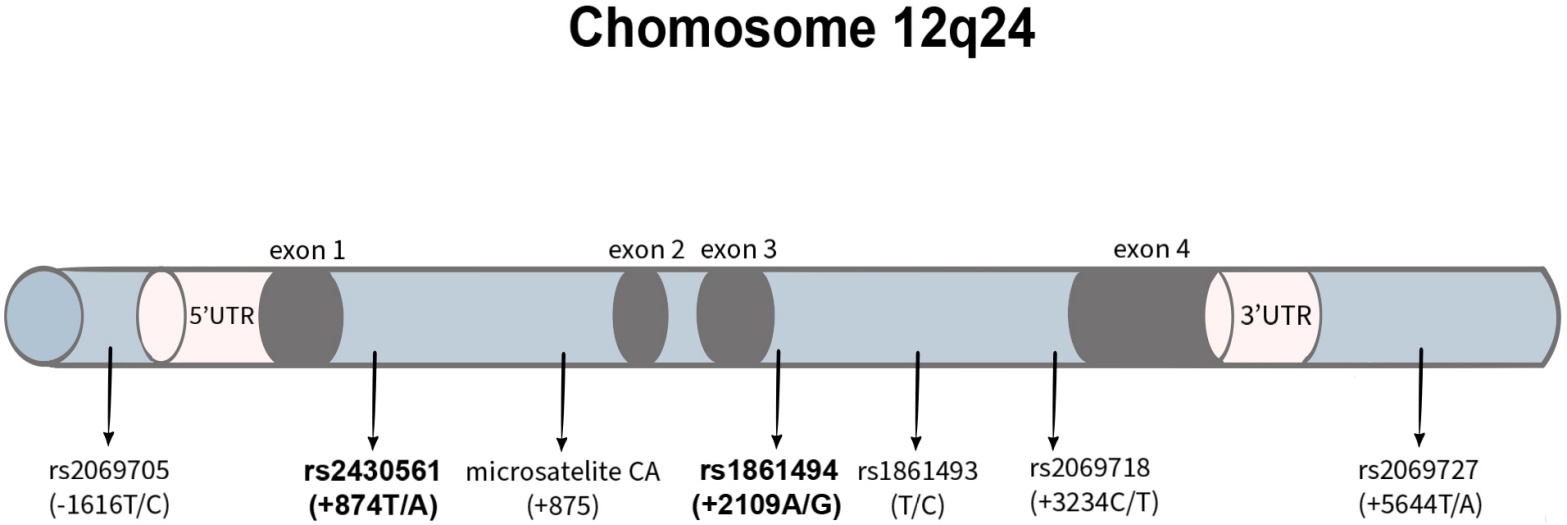
IFN-γ gene polymorphisms with marked variants analyzed in this study.

Genetic factors are known to influence IFN-γ expression. The IFN-γ gene, located on chromosome 12q24, spans approximately 5.4 kb and comprises four exons and three introns (24). Numerous single nucleotide polymorphisms (SNPs) have been identified within this region (12, 24), including the +874T/A (rs2430561) polymorphism in the first intron and the +2109A/G (rs1861494) polymorphism in the third intron, both of which have been shown to influence IFN-γ serum levels (13, 25–27) (SNPs in the gene for IFN-γ are shown in **Figure 2**). Given that IFN-γ function is directly proportional to its concentration, these polymorphisms may modulate immune responses and consequently alter infection outcomes.

It should be emphasized that IFN-γ levels were measured at the time of diagnosis, during active infection. Therefore, the observed differences likely reflect genotype-dependent variability in the magnitude of immune activation rather than constitutive (baseline) cytokine expression. This distinction is important for interpreting the immunogenetic associations observed in this study.

The +874TT genotype of the IFN-γ gene has been shown to be associated with robust IFN-γ production, whereas the AA and TA genotypes correlate with reduced cytokine expression and low IFN-γ production (28). In the present study, we demonstrated significantly lower serum IFN-γ concentrations in patients with the +874AA genotype compared to those with the +874AT and +874TT genotypes. Additionally, we observed a significantly higher frequency of the AA genotype at +874T>A in the serofast group compared to the serologically cured group (44% vs. 10%, p < 0.05). Conversely, the TT genotype, which is associated with higher IFN-γ production, was more prevalent in the serologically cured group (35% vs. 11%, p < 0.05). Similar trends have been reported in other infectious diseases, where the T allele has been linked to stronger immune responses and disease control (29, 30). The protective effect of the T allele has also been documented in TB, where it was associated with milder or localized disease forms (29). Likewise, Wei et al. (31) demonstrated that the T allele of IFN-γ +874T/A (high IFN producer) confers resistance to *Mycobacterium tuberculosis* infection by enhancing IFN-γ expression. The reduced IFN-γ production in these genotypes may contribute to incomplete bacterial clearance, persistent antigenic stimulation, and chronic inflammation, thereby promoting serofast status post-treatment.

Previous studies indicate that the G allele of the IFN-γ rs1861494 polymorphism is associated with lower IFN-γ production, which may influence susceptibility to disease in different ways. In the study from Argentina, individuals carrying the GG genotype exhibited the lowest IFN-γ secretion in response to *Mycobacterium tuberculosis* antigens (8). Compared to AA and AG carriers, GG individuals had significantly reduced levels of IFN-γ in culture supernatants and a lower percentage of CD4^+^IFN-γ^+^ lymphocytes (8). This suggests that the G allele is linked to decreased IFN-γ expression, which may impair the immune response against tuberculosis and contribute to increased disease susceptibility.

**Figure 3:**
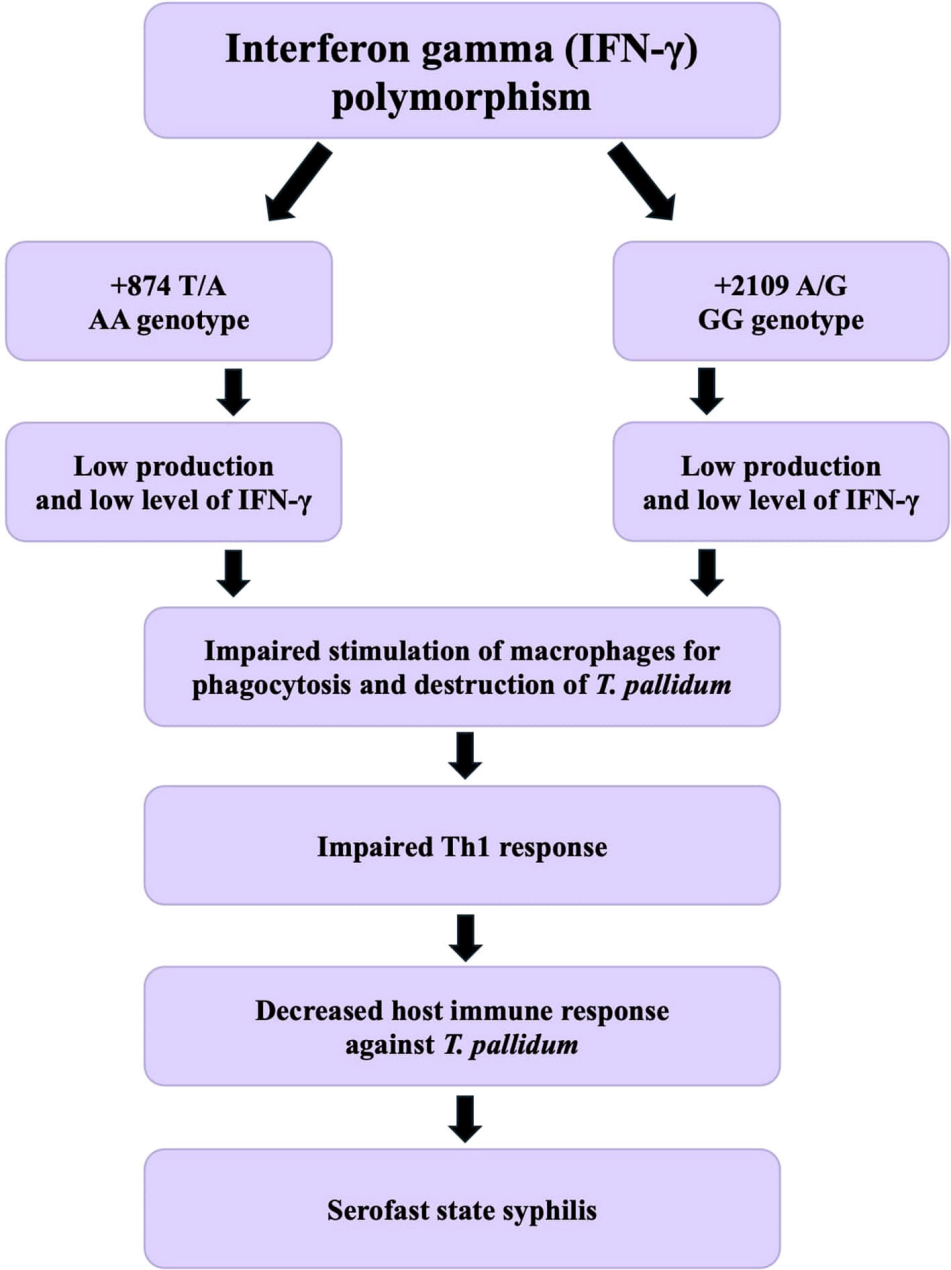
Hypothetical mechanism linking IFN-γ gene polymorphisms to serofast state after syphilis treatment.

**Figure 3** illustrates the authors’ hypothesis that polymorphisms in the IFN-γ gene - specifically the AA genotype of the +874 T/A polymorphism and the GG genotype of the +2109 A/G polymorphism - lead to reduced IFN-γ production. This results in diminished stimulation of macrophages for the phagocytosis and destruction of *T. pallidum*, representing an impairment of the host’s cellular immune response. Consequently, these alterations may predispose individuals to the development of a serofast state following early syphilis treatment.

A meta-analysis evaluating the efficacy of various antibiotics in the treatment of syphilis demonstrated that doxycycline, when compared to benzathine penicillin, is associated with a higher rate of serological failure (32). In addition to their antimicrobial properties, tetracyclines suppress the production of pro-inflammatory cytokines and exert immunomodulatory effects (33–35). Penicillins, on the other hand, disrupt bacterial cell walls, leading to the release of pro-inflammatory components into the host system (36–38). These findings suggest that the choice of antibiotic regimen may influence immunological dynamics and treatment outcomes, potentially interacting with host genetic predispositions.

Despite the strengths of our study, some limitations should be acknowledged. First, the sample size was relatively small, necessitating validation in larger cohorts. Multi-center studies would be beneficial to provide a broader perspective on IFN-γ polymorphisms in syphilis serological outcomes. Second, our study population was homogenous, and genetic variations across different ethnic groups were not assessed. Finally, the observational nature of our study limits causal inferences. Although we identified associations between IFN-γ polymorphisms and serological outcomes, experimental studies or longitudinal assessments with repeated immune profiling could help establish causal relationships between genetic predisposition and serofast status.

## 5 Conclusions

The results of our study introduce a new, previously unexplored direction in research on the phenomenon of serofast state syphilis. The current literature contains a few studies on the polymorphisms of the IFN-γ gene in the pathogenesis of various diseases—primarily viral and bacterial – but to our best knowledge, our study is the first to address the impact of IFN-γ gene polymorphisms in the context of the immune response to *T. pallidum* infection, the persistence of infection, and the occurrence of the serofast state in syphilis. We demonstrated that the +874 T>A and +2109 A>G polymorphisms, involved in many diseases, also influence IFN-γ production in patients with syphilis.

The results of our study indicate a significant role for single nucleotide polymorphisms (SNPs) in the IFN-γ gene and their haplotypes in modulating the immune response in syphilis patients. Our findings suggest that the +874 T>A and +2109 A>G polymorphisms in the IFN-γ gene, as well as the haplotypes associated with them, may influence IFN-γ production levels. We demonstrated that the AA genotype (+874 T>A) and GG genotype (+2109 A>G) were statistically significantly associated with lower serum concentrations of IFN-γ and were linked to the group of patients who develop the serofast state after early syphilis treatment.

These polymorphisms, by reducing IFN-γ production, which is one of the main mediators in the cellular response to *T. pallidum* infection, may result in low serum levels of this cytokine in patients. Consequently, this may contribute to incomplete tissue eradication of the bacteria, prolongation of inflammation, and the occurrence of serological resistance after syphilis treatment. Our observation suggests that these polymorphisms are a genetic risk factor for the development of the serofast state.

The analysis of these polymorphisms may be useful in assessing the risk of serofast status in syphilis patients. These polymorphisms could become prognostic biomarkers, enabling the personalization of diagnostic and therapeutic approaches in the future.

Further studies on larger patient groups are necessary to validate our findings and to assess the significance of genetic and immunological factors in the development of the serofast state. In the future, it may be possible to harness the therapeutic potential of modulating pathways associated with IFN-γ in individuals who develop the serofast state.

## Data Availability

The raw data supporting the conclusions of this article will be made available by the authors, without undue reservation.

## References

[1] Workowski KA Berman S Centers for Disease Control and Prevention (CDC). Sexually transmitted diseases treatment guidelines, 2010. MMWR Recomm Rep. (2010) 59:1–110. 21160459

[2] Workowski KA Bolan GA. Sexually transmitted diseases treatment guidelines, 2015. MMWR Recomm Rep. (2015) 64:1–137. PMC5885289 26042815

[3] Pastuszczak M Gozdzialska A Jakiela B Obtulowicz A Jaskiewicz J Wojas-Pelc A. Robust pro-inflammatory immune response is associated with serological cure in patients with syphilis: an observational study. Sex Transm Infect. (2017) 93:11–4. doi: 10.1136/sextrans-2016-052681 27356549

[4] Pastuszczak M Jakiela B Wojas-Pelc A. Association of interleukin-10 promoter polymorphisms with serofast state after syphilis treatment. Sex Transm Infect. (2019) 95:163–8. doi: 10.1136/sextrans-2018-053753 30341234

[5] Zhang RC Zheng NN Zhong LS. Association of TNF-α -308 G>A gene polymorphism with serofast in patients with syphilis. Australas J Dermatol. (2021) 62:e294–6. doi: 10.1111/ajd.13481 33200819

[6] Esparza Guerrero Y Vazquez Villegas ML Nava Valdivia CA Ponce Guarneros JM Perez Guerrero EE Gomez Ramirez EE. Association of the STAT4 gene rs7574865 polymorphism with IFN-γ Levels in patients with systemic lupus erythematosus. Genes. (2023) 14:537. doi: 10.3390/genes14030537 36980810 PMC10048585

[7] de Sarges KML Póvoa da Costa F Santos EFD Cantanhede MHD da Silva R Veríssimo ADOL. Association of the IFNG +874T/A polymorphism with symptomatic COVID-19 susceptibility. Viruses. (2024) 16:650. doi: 10.3390/v16040650 38675991 PMC11053931

[8] Rolandelli A Pellegrini JM Amiano NO Santilli MC Morelli MP Castello FA. The IFNG rs1861494 Single Nucleotide Polymorphism Is Associated with Protection against Tuberculosis Disease in Argentina. Genes (Basel). (2018) 9:46. doi: 10.3390/genes9010046 29361774 PMC5793197

[9] Gao J Wei L Liu X Wang L Niu D Jin T. Association between IFN-γ Gene polymorphisms and igA nephropathy in a chinese han population. Kidney Blood Press Res. (2017) 42:136–44. doi: 10.1159/000473889 28391282

[10] Zheng M Li J Fang W Luo L Ding R Zeng H. The TNF-α rs361525 and IFN-γ rs2430561 polymorphisms are associated with liver cirrhosis risk: a comprehensive meta-analysis. Front Immunol. (2023) 14:1129767. doi: 10.3389/fimmu.2023.1129767 37122734 PMC10140545

[11] Ahmed AA Rasheed Z Salem T Al-Dhubaibi MS Al Robaee AA Alzolibani AA. TNF-α -308 G/A and IFN-γ +874 A/T gene polymorphisms in Saudi patients with cutaneous leishmaniasis. BMC Med Genet. (2020) 21:104. doi: 10.1186/s12881-020-01043-9 32404058 PMC7218653

[12] Dondeti MF Abdelkhalek MS El-Din Elezawy HM Alsanie WF Raafat BM Gamal-Eldeen AM. Association between interferon-gamma (IFN-γ) gene polymorphisms (+874A/T and +2109A/G), and susceptibility to hepatitis B viral infection (HBV). J Appl Biomed. (2022) 20:37–43. doi: 10.32725/jab.2022.001 35099129

[13] Chevillard C Moukoko CE Elwali NE Bream JH Kouriba B Argiro L. IFN-gamma polymorphisms (IFN-gamma +2109 and IFN-gamma +3810) are associated with severe hepatic fibrosis in human hepatic schistosomiasis (Schistosoma mansoni). J Immunol. (2003) 171:5596–601. doi: 10.4049/jimmunol.171.10.5596 14607968

[14] Workowski KA Bachmann LH Chan PA Johnston CM Muzny CA Park I. Sexually transmitted infections treatment guidelines, 2021. MMWR Recomm Rep. (2021) 70:1–187. doi: 10.15585/mmwr.rr7004a1 PMC8344968 34292926

[15] Pastuszczak M Wojas-Pelc A. Current standards for diagnosis and treatment of syphilis: selection of some practical issues, based on the European (IUSTI) and U.S. (CDC) guidelines. Postepy Dermatol Alergol. (2013) 30:203–10. doi: 10.5114/pdia.2013.37029 PMC3834708 24278076

[16] Seña AC Zhang XH Li T Zheng HP Yang B Yang LG. A systematic review of syphilis serological treatment outcomes in HIV-infected and HIV-uninfected persons: rethinking the significance of serological non-responsiveness and the serofast state after therapy. BMC Infect Dis. (2015) 15:479. doi: 10.1186/s12879-015-1209-0 26511465 PMC4625448

[17] Kaminiów K Kiołbasa M Pastuszczak M. The significance of the cell-mediated host immune response in syphilis. Microorganisms. (2024) 12:2580. doi: 10.3390/microorganisms12122580 39770782 PMC11677580

[18] Leader BT Godornes C VanVoorhis WC Lukehart SA. CD4+ lymphocytes and gamma interferon predominate in local immune responses in early experimental syphilis. Infect Immun. (2007) 75:3021–6. doi: 10.1128/IAI.01973-06 PMC1932874 17403876

[19] Van Voorhis WC Barrett LK Koelle DM Nasio JM Plummer FA Lukehart SA. Primary and secondary syphilis lesions contain mRNA for Th1 cytokines. J Infect Dis. (1996) 173:491–5. doi: 10.1093/infdis/173.2.491 8568320

[20] Hernández-Pliego A Vergara-Ortega DN Herrera-Ortíz A Toledano-Jaimes C Esquivel-Guadarrama FR Sánchez-Alemán MÁ. IL-10 and IL-17 as progression markers of syphilis in people living with HIV: A systematic review. Biomolecules. (2022) 12:1472. doi: 10.3390/biom12101472 36291681 PMC9599307

[21] Li W Wu W Chang H Jiang M Gao J Xu Y. Cerebrospinal fluid cytokines in patients with neurosyphilis: the significance of interleukin-10 for the disease. BioMed Res Int. (2020) 2020:3812671. doi: 10.1155/2020/3812671 33083463 PMC7556108

[22] Hawley KL Cruz AR Benjamin SJ La Vake CJ Cervantes JL LeDoyt M. IFNγ Enhances CD64-potentiated phagocytosis of treponema pallidum opsonized with human syphilitic serum by human macrophages. Front Immunol. (2017) 8:1227. doi: 10.3389/fimmu.2017.01227 29051759 PMC5633599

[23] Li J Wang LN Zheng HY. Predictors of serological cure and serofast state after treatment in HIV-negative patients with early syphilis in China. Sex Transm Infect. (2013) 89:69. doi: 10.1136/sextrans-2012-050711 22914681

[24] Sun Y Lu Y Li T Xie L Deng Y Li S. Interferon gamma +874T/A polymorphism increases the risk of hepatitis virus-related diseases: evidence from a meta-analysis. PloS One. (2015) 10:e0121168. doi: 10.1371/journal.pone.0121168 25939029 PMC4418602

[25] Peresi E Oliveira LR da Silva WL da Costa EA Araujo JP Jr Ayres JA. Cytokine polymorphisms. Their influence and levels in Brazilian patients with pulmonary tuberculosis during antituberculosis treatment. Tuberc Res Treat. (2013) 2013:285094. doi: 10.1155/2013/285094 23634300 PMC3619634

[26] Pravica V Perrey C Stevens A Lee JH Hutchinson IV. A single nucleotide polymorphism in the first intron of the human IFN-gamma gene: absolute correlation with a polymorphic CA microsatellite marker of high IFN-gamma production. Hum Immunol. (2000) 61:863–6. doi: 10.1016/s0198-8859(00)00167-1 11053629

[27] Henri S Stefani F Parzy D Eboumbou C Dessein A Chevillard C. Description of three new polymorphisms in the intronic and 3’UTR regions of the human interferon gamma gene. Genes Immun. (2002) 3:1–4. doi: 10.1038/sj.gene.6363809 11857052

[28] Schena FP Cerullo G Torres DD Scolari F Foramitti M Amoroso A. Role of interferon-gamma gene polymorphisms in susceptibility to IgA nephropathy: a family-based association study. Eur J Hum Genet. (2006) 14:488–96. doi: 10.1038/sj.ejhg.5201591 16493441

[29] Wani BA Shehjar F Shah S Koul A Yusuf A Murtaza M. Association of IFN-gamma and IL-10 gene variants with the risk of extrapulmonary tuberculosis. Saudi J Biol Sci. (2021) 28:4210–6. doi: 10.1016/j.sjbs.2021.06.029 PMC8324987 34354401

[30] Sousa-Vasconcelos PS Seguins WS Luz ES Pinho RT. Pattern of cytokine and chemokine production by THP-1 derived macrophages in response to live or heat-killed Mycobacterium bovis bacillus Calmette-Guérin Moreau strain. Mem Inst Oswaldo Cruz. (2015) 110:809–13. doi: 10.1590/0074-02760140420 PMC4667587 26517663

[31] Wei Z Wenhao S Yuanyuan M Yang L Daming Z Jiangchun X. A single nucleotide polymorphism in the interferon-γ gene (IFNG +874 T/A) is associated with susceptibility to tuberculosis. Oncotarget. (2017) 8:50415–29. doi: 10.18632/oncotarget.17304 PMC5584145 28881572

[32] Liu HY Han Y Chen XS Bai L Guo SP Li L. Comparison of efficacy of treatments for early syphilis: A systematic review and network meta-analysis of randomized controlled trials and observational studies. PloS One. (2017) 12:e0180001. doi: 10.1371/journal.pone.0180001 28658325 PMC5489196

[33] Sun J Shigemi H Tanaka Y Yamauchi T Ueda T Iwasaki H. Tetracyclines downregulate the production of LPS-induced cytokines and chemokines in THP-1 cells via ERK, p38, and nuclear factor-κB signaling pathways. Biochem Biophys Rep. (2015) 4:397–404. doi: 10.1016/j.bbrep.2015.11.003 29124230 PMC5669446

[34] Di Caprio R Lembo S Di Costanzo L Balato A Monfrecola G. Anti-inflammatory properties of low and high doxycycline doses: an *in vitro* study. Mediators Inflamm. (2015) 2015:329418. doi: 10.1155/2015/329418 25977597 PMC4421036

[35] Orylska-Ratynska M Placek W Owczarczyk-Saczonek A. Tetracyclines—An important therapeutic tool for dermatologists. Int J Environ Res Public Health. (2022) 19:7246. doi: 10.3390/ijerph19127246 35742496 PMC9224192

[36] Moore LJ Pridmore AC Lee ME Read RC. Induction of pro-inflammatory cytokine release by human macrophages during exposure of Streptococcus pneumoniae to penicillin is influenced by minimum inhibitory concentration ratio. Int J Antimicrob Agents. (2005) 26:188–96. doi: 10.1016/j.ijantimicag.2005.06.006 16099623

[37] Wolf AJ Liu GY Underhill DM. Inflammatory properties of antibiotic-treated bacteria. J Leukoc Biol. (2017) 101:127–34. doi: 10.1189/jlb.4MR0316-153RR PMC5166437 27576461

[38] Gross JL Basu R Bradfield CJ Sun J John SP Das S. Bactericidal antibiotic treatment induces damaging inflammation via TLR9 sensing of bacterial DNA. Nat Commun. (2024) 15:10359. doi: 10.1038/s41467-024-54497-3 39609397 PMC11605096

